# A smoking-associated 7-gene signature for lung cancer diagnosis and prognosis

**DOI:** 10.3892/ijo.2012.1556

**Published:** 2012-07-16

**Authors:** YING-WOOI WAN, REBECCA A. RAESE, JAMES E. FORTNEY, CHANGCHANG XIAO, DAJIE LUO, JOHN CAVENDISH, LAURA F. GIBSON, VINCENT CASTRANOVA, YONG QIAN, NANCY LAN GUO

**Affiliations:** 1 Mary Randolph Cancer Center;; 2 Departments of Community Medicine; 3 Statistics and; 4 Microbiology, Immunology and Cell Biology, West Virginia University, Morgantown, WV 26506;; 5 Pathology and Physiology Research Branch, Health Effects Laboratory Division, National Institute for Occupational Safety and Health, Morgantown, WV 26505, USA

**Keywords:** smoking, lung cancer diagnosis and prognosis, gene signature, signaling pathway, coexpression networks

## Abstract

Smoking is responsible for 90% of lung cancer cases. There is currently no clinically available gene test for early detection of lung cancer in smokers, or an effective patient selection strategy for adjuvant chemotherapy in lung cancer treatment. In this study, concurrent coexpression with multiple signaling pathways was modeled among a set of genes associated with smoking and lung cancer survival. This approach identified and validated a 7-gene signature for lung cancer diagnosis and prognosis in smokers using patient transcriptional profiles (n=847). The smoking-associated gene coexpression networks in lung adenocarcinoma tumors (n=442) were highly significant in terms of biological relevance (network precision = 0.91, FDR<0.01) when evaluated with numerous databases containing multi-level molecular associations. The gene coexpression network in smoking lung adenocarcinoma patients was confirmed in qRT-PCR assays of the identified biomarkers and involved signaling pathway genes in human lung adenocarcinoma cells (H23) treated with 4-(methylnitrosamino)-1-(3-pyridyl)-1-butanone (NNK). Furthermore, the western blotting results of *p53*, phospho-*p53*, *Rb* and *EGFR* in NNK-treated H23 and transformed normal human lung epithelial cells (BEAS-2B) support their functional involvement in smoking-induced lung cancer carcinogenesis and progression.

## Introduction

Lung cancer remains the leading cause of cancer-related mortality for both men and women, and its incidence is increasing worldwide (1). Smoking is the strongest population-attributable risk factor in lung carcinogenesis and is responsible for approximately 90% of lung cancer incidents (2–4). Currently, there are no effective diagnostic screening tools for early detection of lung cancer in smokers. CT scans are offered for lung cancer screening in smokers. Nevertheless, neither the American Cancer Society nor the U.S. Preventive Services Task Force recommends CT scans due to concerns about accuracy in the interpretation of results. Furthermore, the mechanistic effect of smoking on lung cancer progression remains unclear. Despite our previous finding that smoking intensity at the time of diagnosis is a significant and independent prognostic factor for lung cancer (5), smoking status in itself is not a prognostic determinant of lung cancer.

Non-small cell lung cancer (NSCLC) accounts for 85–90% of lung cancer cases. NSCLC includes two major subtypes, adenocarcinoma and squamous cell carcinoma. Owing to the limitations of the current screening techniques, most patients with NSCLC are diagnosed at advanced disease stage. A minority (∼25–30%) of patients with NSCLC are diagnosed with stage I disease and receive surgical resection as the major treatment option (6). However, 35–50% of stage I NSCLC patients will relapse within five years following surgery (6), indicating that a subgroup of these patients might benefit from adjuvant chemotherapy. Meanwhile, adjuvant chemotherapy of stage II and stage III disease has resulted in only modest survival benefits (7). While tumor recurrence remains the major treatment failure for lung cancer, it is not currently possible to identify specific high-risk patients for adjuvant chemotherapy. As a consequence, current multi-modality therapy is of limited efficacy, with an overall 5-year survival rate of about 15% (8).

In this study, we sought to identify a gene signature for lung cancer diagnosis and prognosis in smokers. Genes implicated in cancer initiation and progression show dysregulated interactions with their molecular partners (9), and these cancer genes are more likely to actively interact with signaling proteins (10). Because tumors utilize different signaling pathways, we modeled crosstalk with a diverse set of signaling pathways to identify gene signatures that perform more uniformly across heterogeneous tumor sets. Specifically, implication networks (11,12) were used to model concurrent coexpression with multiple signaling pathways among a set of genes associated with smoking and lung cancer survival. This approach identified and validated a smoking-associated 7-gene signature using patient microarray profiles of (n=847). Furthermore, BEAS-2B cell line transformed from normal human lung epithelial cells and human lung adenocarcinoma cells (H23) were treated with 4-(methylnitrosamino)-1-(3-pyridyl)-1-butanone (NNK), a major tobacco-specific carcinogen (13,14), for qRT-PCR and western blots validation of the identified biomarkers and involved signaling pathways.

## Materials and methods

### Microarray profiles and patient samples

Four patient cohorts with published microarray transcriptional profiles were used in this study. The first cohort contains 442 lung adenocarcinoma patient samples from the Director’s Challenge Study (15). This study cohort is composed of 4 data sets (University of Michigan, H. Lee Moffitt Cancer Center, Memorial Sloan-Kettering Cancer Center and Dana-Farber Cancer Institute) contributed by 6 institutions. The clinical characteristics and smoking status of the patients are summarized in Table I.

The second patient cohort contains 130 squamous cell lung cancer samples from Raponi *et al*(16). The third cohort contains 111 NSCLC samples from Bild *et al*(17). The fourth cohort contains 164 airway epithelial cell lung tissue samples from current and former smokers published by Spira *et al*(2). This cohort has 60 lung cancer samples (48 NSCLC, 11 small cell lung cancer and 1 unknown histology) and 69 normal lung tissue samples. Patient gene expression profiles from Shedden *et al*(15), Raponi *et al*(16) and Spira *et al*(2) were quantified with Affymetrix HG-U133A. The dataset from Bild *et al*(17) was quantified with Affymetrix HG-U133 Plus 2. The raw microarray data were quantile-normalized and log2 transformed with dChip (18) for further analysis.

### Implication networks

The implication induction algorithm (11) based on prediction logic (19) was used to derive coexpression between each pair of genes using software Genet (11,12,20). In the biological context, the six foremost implication rules relating two dichotomous variables are interpreted as follows: *A* ⇒ *B*: upregulation of gene *A* causes upregulation of gene *B; A* ⇒ ¬*B*: upregulation of gene *A* causes downregulation of gene *B*; ¬*A* ⇒ *B*: downregulation of gene *A* causes upregulation of gene *B*; ¬*A* ⇒ ¬*B*: downregulation of gene *A* causes downregulation of gene *B; A* ⇔ *B*: upregulation of gene *A* causes upregulation of gene *B* and upregulation of gene *B* causes upregulation of gene *A; A* ⇔ ¬*B*: upregulation of gene *A* causes downregulation of gene *B* and downregulation of gene *B* causes upregulation of gene *A*. Mean expression of each gene in the training set was used to define up- or downregulation. The minimum scope and the minimum precision of a derived implication relation were significantly greater than zero (P<0.05, one-sided z-tests).

### Evaluation of gene coexpression networks

The following pathway databases were used to evaluate the biological relevance of the derived coexpression networks, including NCBI Entrez Gene (21), Kyoto Encyclopedia of Genes and Genomes (KEGG) (22), NCI-Nature Pathway Interaction Database (http://pid.nci.nih.gov/), protein-protein interaction database STRING 8 (23), and Pathway Studio 7.0 (Ariadne Genomics, Rockville, MD, USA). In addition, five gene set collections [positional, curated, motif, computational and Gene Oncology (GO)] and canonical pathway databases from the MSigDB (24) were used in the network precision and FDR evaluation. Using these resources, a coexpression relation is considered a true positive (TP) if the pair of genes satisfy any of the following: i) on the same chromosome or cytogenetic band; ii) in the same curated or canonical pathway; iii) sharing a cis-regulator motif, binding motif, or transcription factor binding site; iv) annotated by the same GO term; v) having protein-protein interaction; or vi) within the same computational gene sets mined from cancer-oriented microarray data. The coexpression relation is considered a false positive (FP) if the gene pair do not satisfy all five conditions listed above (25). If at least one gene in the pair is not annotated, a coexpression relation is labeled as non-discriminatory (ND). Coexpression relations labeled as ND were excluded in this evaluation as they were not confirmed. Network precision is defined as:

$$\mathrm{network}\_\mathrm{precision}=\frac{\mathrm{TP}}{\mathrm{TP}+\mathrm{FP}}$$

The portion of FP over all positive cases is defined as q-value:

$$q–\mathrm{value}=\frac{\mathrm{FP}}{\mathrm{TP}+\mathrm{FP}}$$

The FDR of the smoking-mediated coexpression networks was calculated by averaging the q-values obtained from the null distribution generated in 1,000 random permutations of the class labels in the test cohort.

The stability of the computationally derived smoking-mediated coexpression networks was evaluated using different subsets of patient samples from the training set in 100 iterations. The stability is defined as the portion of the smoking-mediated coexpression relations obtained from the original data that were retrieved by using only a random subset of the training data and the full test data.

### Cell cultures

NCI-H23 (ATCC no. CRL-5800) cells were cultured in RPMI-1640 medium (Mediatech, Manassas, VA, USA) supplemented with 10% FBS (Hyclone, Logan, UT, USA), 2 mM L-glutamine (Mediatech), 100 IU penicillin/ml (Sigma, St. Louis, MO, USA), and 100 μg streptomycin/ml (Sigma). BEAS-2B (ATCC no. CRL-9609) cells were cultured in Dulbecco’s modified Eagle’s medium (Mediatech) supplemented with 5% FBS (Hyclone), 2 mM L-glutamine (Mediatech), 100 IU penicillin/ml (Sigma) and 100 μg streptomycin/ml (Sigma).

### NNK treatment and protein isolation

H23 and BEAS-2B cells were treated with 100 nM NNK (Toronto Research Chemicals, North York, ON, Canada) for 15 min, 1 and 16 h. Four repeats (total of five samples) were performed on each cell line and for each time point. Following treatment, cells were harvested by trypsinization and protein was isolated. Cells were lysed in CLB lysis buffer (50 mM Tris-HCl, pH 7.4, 150 mM NaCl, 1% Triton X-100, 0.25% Na-deoxycholate, 5 mM EDTA and 1 mM NaF) supplemented with 1% (v/v) HALT Protease Inhibitor Cocktail, purchased from Thermo Scientific (Rockford, IL, USA), on ice for 15 min with occasional mixing by vortex. Lysates were centrifuged at 20,800 × g for 15 min to pellet insoluble debris and then supernatants were collected. Total protein concentration was determined by bicinchonic acid (BCA) protein assay purchased from Pierce Protein Research Products (Rockford, IL, USA).

### SDS-PAGE and western blotting

Proteins (50 μg) were resolved on precast Mini-Protean TGX Gels (4–20%; Bio-Rad Laboratories, Hercules, CA, USA). After boiling for 5 min with reducing Laemmli buffer, proteins were separated and subsequently transferred to a PVDF membrane at 100 mV for 1 h at 4°C. After transfer, membranes were blocked in NET-gelatin solution (150 mM NaCl, 5 mM EDTA, 50 mM Tris-HCl, pH 7.5, 0.05% Triton X-100 and 0.25% gelatin) for 1 h at room temperature. Primary antibody was added to membranes in 15 ml NET-gelatin solution [1:500 dilution for anti-EGFR, 1:25,000 dilution for anti-GAPDH, 1:2,000 dilution for anti-p53, 1:1,000 dilution for phospho-p53 (phospho S15) and anti-Rb] and membranes were incubated for 2 h at room temperature with rocking. Membranes were then washed in NET-gelatin solution (3 x 20 min with shaking) with HRP-conjugated secondary monoclonal anti-mouse IgG antibody purchased from GE Healthcare UK Ltd. (Little Chalfont, UK). After 1 h of incubation, unbound secondary antibody was removed by washing in NET-gelatin solution (3 x 20 min with shaking). Signal was visualized using Immobilon chemiluminescent HRP substrate from Millipore (Billerica, MA, USA). Primary antibodies utilized included mouse monoclonal anti-EGFR, from Thermo Fisher Scientific (Fremont, CA, USA), and mouse monoclonal anti-GAPDH purchased from Fitzgerald Industries International Inc. (Acton, MA, USA). In addition, the following antibodies were used in western blotting: anti-p53 [Abcam, Mouse Monoclonal (ab26)], anti-phospho-p53/phospho S15 [Abcam, Rabbit Polyclonal (ab1431)] and anti-Rb [Abcam, Mouse Monoclonal (ab24)].

### Densitometry

Relative *EGFR*, *p53*, phospho-*p53*, and *Rb* expression was determined by densitometric analysis using ImageJ software provided by NIH (http://rsb.info.nih.gov/ij/index.html). X-ray films were scanned at 300 and 600 DPI using a CanoScan (Canon, Lake Success, NY, USA) and images were imported into ImageJ for analysis. The raw signal intensity was determined by selecting the peak corresponding to each band and integrating the intensity within that peak. Local background intensity (calculated by averaging the background intensities at the upper and lower bounds of the peak) was integrated and subtracted from each raw intensity to give the background-corrected signal intensity. To account for loading differences, the corrected signal intensity for the assayed proteins was divided by the corrected GAPDH intensity. Samples treated with varying NNK exposure times were compared to untreated controls for the H23 and BEAS-2B cell lines.

### RNA isolation, complementary DNA synthesis, and qRT-PCR gene expression profiling

Total-RNA was isolated from both cell lines using the mirVana™ miRNA Isolation kit and following the manufacturer’s protocol (Ambion, Austin, TX, USA). Total-RNA was eluted in 100 μl of nuclease-free water and stored at −80°C. RNA concentration was determined using the NanoDrop 1000 Spectrophotometer (NanoDrop Technologies, Wilmington, DE, USA). RNA quality, 28S/18S ratio, and a visual image of the 28S and 18S bands were evaluated using the 2100 Bioanalyzer (Agilent Technologies, Santa Clara, CA, USA). Total-RNA (1 μg) was converted into complementary DNA (cDNA) using the High Capacity cDNA Reverse Transcription Kit from Applied Biosystems (Life Technologies, Carlsbad, CA, USA). Thermal cycling conditions were as follows: 25°C for 10 min, 2 cycles of 37°C for 60 min and 85°C for 5 sec followed by a programmed hold at 4°C.

All qRT-PCR reactions were performed on a 7500 real-time PCR system from Applied Biosystems. The reports were generated using SDS2.3 software (Applied Biosystems). The Ct values obtained were normalized to the UBC housekeeping gene in each sample. Fold changes were computed using the 2^−ΔΔ*Ct*^ method of 5 biological replicates and 3 technical replicates (26). Statistical significance was computed using repeated ANOVA tests in *R* and is considered statistically significant at P≤0.05.

The coexpression relation of a gene pair derived with the implication induction algorithm was compared with the observed NNK-induced gene expression changes. The coexpression relation is confirmed when the observed NNK-induced gene expression changes are consistent with the predicted implication rule between the two genes. For example, if the rule between gene A and gene B is positive equivalence (A⇔B), it is confirmed if both gene A and gene B showed overexpression in the corresponding experiments.

## Results

### Identification of a smoking-associated 7-gene signature

Lung cancer survival genes were first selected from the whole genome on the training set (UM and HLM; n=256) from Shedden *et al*(15). A total of 2,310 genes were significantly associated with overall survival (P<0.05, univariate Cox model). Second, from this set of 2,310 genes, 217 genes exhibited significant differential expression (P<0.05, t-tests) in smokers versus non-smokers. These 217 survival and smoking-associated genes, as well as 6 major signaling pathway genes (*EGF*, *EGFR*, *MET*, *KRAS*, *E2F3*, and *E2F5*) were included in the network analysis. These signaling pathways are included in human NSCLC disease mechanisms delineated by the KEGG Pathway Database (http://www.genome.jp/kegg/pathway/hsa/hsa05223.html). They were selected based on their reported clinical relevance in NSCLC. These 6 signaling pathway genes were not significantly associated with survival nor were they differentially expressed in smokers.

**Figure 1:**
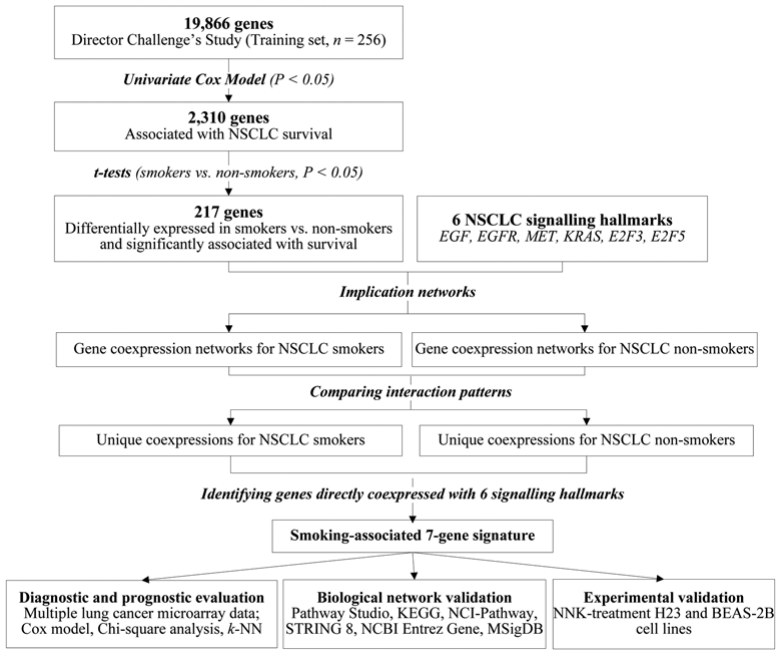
Methodology for network-based identification of smoking-associated 7-gene signature.

Patient samples in the training set were separated into two groups: smokers (patients who smoked in the past or who are currently smoking) and non-smokers (patients who never smoked). For each smoking-defined patient group, a coexpression network among the 223 genes was constructed. Between each pair of the 223 genes, significant (P<0.05; z-tests) coexpression relations were retrieved in the smoker group and the non-smoker group, constituting smoking-mediated gene coexpression networks in NSCLC. By comparing the coexpression types between each pair of genes in the two networks, differential network components were identified and considered important for further evaluation. These differential components are interactions that were present in the smoker group but missing in the non-smoker group, or conversely, those present in the non-smoker group but absent in the smoker group. From the differential components associated with the smoker group and non-smoker group, genes having direct coexpression relations with all 6 lung cancer signaling pathway genes were identified as the signature genes (Fig. 1). As a result, 6 genes were identified from the smoker group and 1 gene was identified from the non-smoker group. This constituted the smoking-associated 7-gene signature for NSCLC (Table II).

### Prognostic validation in lung adenocarcinoma

**Figure 2:**
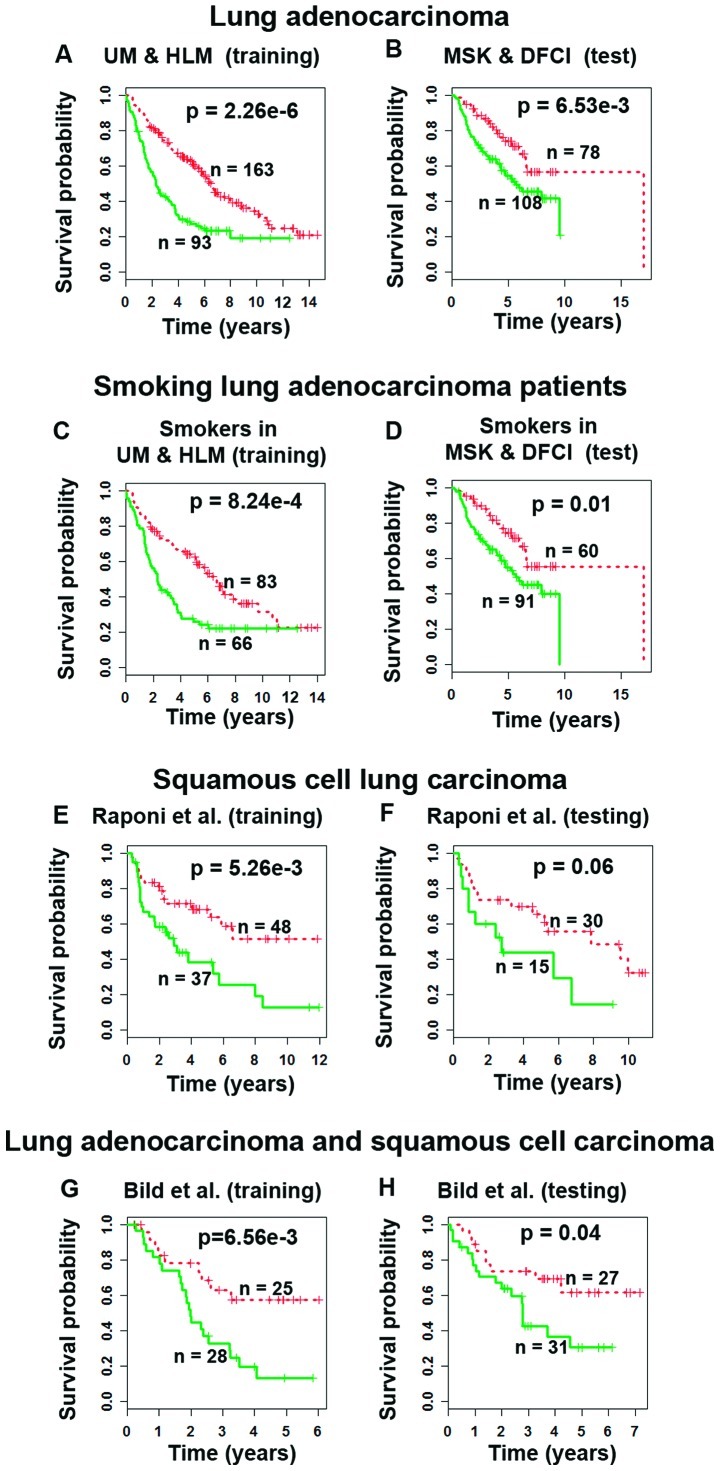
Prognosis in NSCLC patients using smoking-associated 7-gene signature. In the cohorts from Shedden *et al*(15), the risk score giving the best prediction on the 3-year ROC curve generated significant patient stratification (log-rank P<0.007) on the (A) training set and (B) independent test set. This classifier also stratified smoking lung adenocarcinoma patients into two distinct (log-rank P<0.01) prognostic groups in both the (C) training and (D) test sets. Significant stratifications were also obtained in the randomly partitioned training and test sets of patients with squamous cell carcinoma from (E and F) Raponi *et al*(16) and (G and H) the Bild cohort (17) of lung adenocarcinoma and squamous cell carcinoma. Log-rank tests were used to assess the statistical significance in survival probability between the two prognostic groups. Red curves, low-risk patient group; green curves, high-risk patient group.

We sought to investigate if the identified gene signature could provide accurate prognostic prediction of survival in lung adenocarcinoma patients. On the training cohort, the original microarray gene expression profiles of the identified 7 gene probes were fitted into a Cox model as covariates. A survival risk score was generated for each patient in the training set. A training model (Fig. 2A) was identified and applied to the test set (MSK and DFCI; n=186; Fig. 2B) without re-estimation of parameters. In both training and test sets, this scheme separated patients into two groups with different survival outcomes (P<0.007, Kaplan-Meier analyses). The hazard ratio of the 7-gene risk score [HR=1.89, 95% CI: (1.06, 3.38)] was higher than other lung cancer prognostic factors except cancer stage in the test set (Table III). There was no significant difference in prognostic value between the hazard ratio of the 7-gene risk score and cancer stage (II vs. I). The results demonstrate that the 7-gene risk score could provide a more accurate prognosis than some commonly used clinicopathological parameters.

The 7-gene signature gave accurate prognostic prediction in smokers in both training and test sets in Shedden’s cohorts (15) (P<0.01; Fig. 2C and D), but not in non-smokers in Kaplan-Meier analyses (P<0.12, results not shown). In addition, gene expression-defined high- and low-risk groups had significant association with smoking (P<0.02, χ^2^ tests) and smoking cessation (P<0.00001, χ^2^ tests; Table I). These results further confirmed the smoking association of the identified 7-gene signature.

### Prognostic validation on other histological subtypes of NSCLC

The prognostic performance of the 7-gene signature was further evaluated on cohorts from Raponi *et al*(16) and Bild *et al*(17), which include another major subtype of NSCLC, squamous cell lung carcinoma. For robust validation, patient samples in these two studied cohorts were randomly partitioned into separate training and test sets. A prognostic classifier was constructed on the training set using the Cox model and validated on the test set without re-estimation of parameters.

In the Raponi cohort (16) of squamous cell carcinoma patients, the 7-gene signature stratified patients into two groups with distinct survival outcomes (log-rank P<0.005, Kaplan-Meier analysis) in the training set (Fig. 2E). This model generated borderline significant stratification (P=0.06, Kaplan-Meier analysis) in the test set (Fig. 2F). This might be owing to the fact that 10 patients (8%) of Raponi’s cohort were either non-smokers or their smoking status was not known, whereas the 7-gene signature provides refined prognosis specifically in smoking lung cancer patients.

In the Bild cohort (17) containing both lung adenocarcinoma and squamous cell carcinoma patients, the 7-gene signature stratified patients into two distinct survival groups in both training and test sets (P<0.04, Kaplan-Meier analyses) (Fig. 2G and H). Overall, these results demonstrate that the 7-gene signature could select high-risk NSCLC patients with a smoking history for chemotherapy.

### Early diagnostic detection of lung cancer in smokers

We further investigated whether the 7-gene signature could be used for diagnostic screening of lung cancer in smokers. The smoking cohort from Spira *et al*(2) was separated into a training set (n=77) and two independent test sets (n=52 and n=35). Using a nearest neighbour algorithm implemented in WEKA (27), the 7-gene classifier could accurately identify lung cancer patients from normal patients with an overall accuracy of 73 and 74% in two test sets, respectively. The odds ratio of predicted lung cancer risk was highly significant in all three sets [OR=3.85, 95% CI: (1.45, 10.20), P<0.007 in training set; OR=7.35, 95% CI: (2.16, 25.04), P<0.001 in Test set 1; OR=8.45, 95% CI: (1.84, 38.75), P<0.006 in Test set 2; Table II]. These results indicate that the identified 7-gene signature has important implications in diagnostic screening of lung cancer risk in smokers.

### NNK-induced gene and protein expression in BEAS-2B and H23

**Figure 3:**
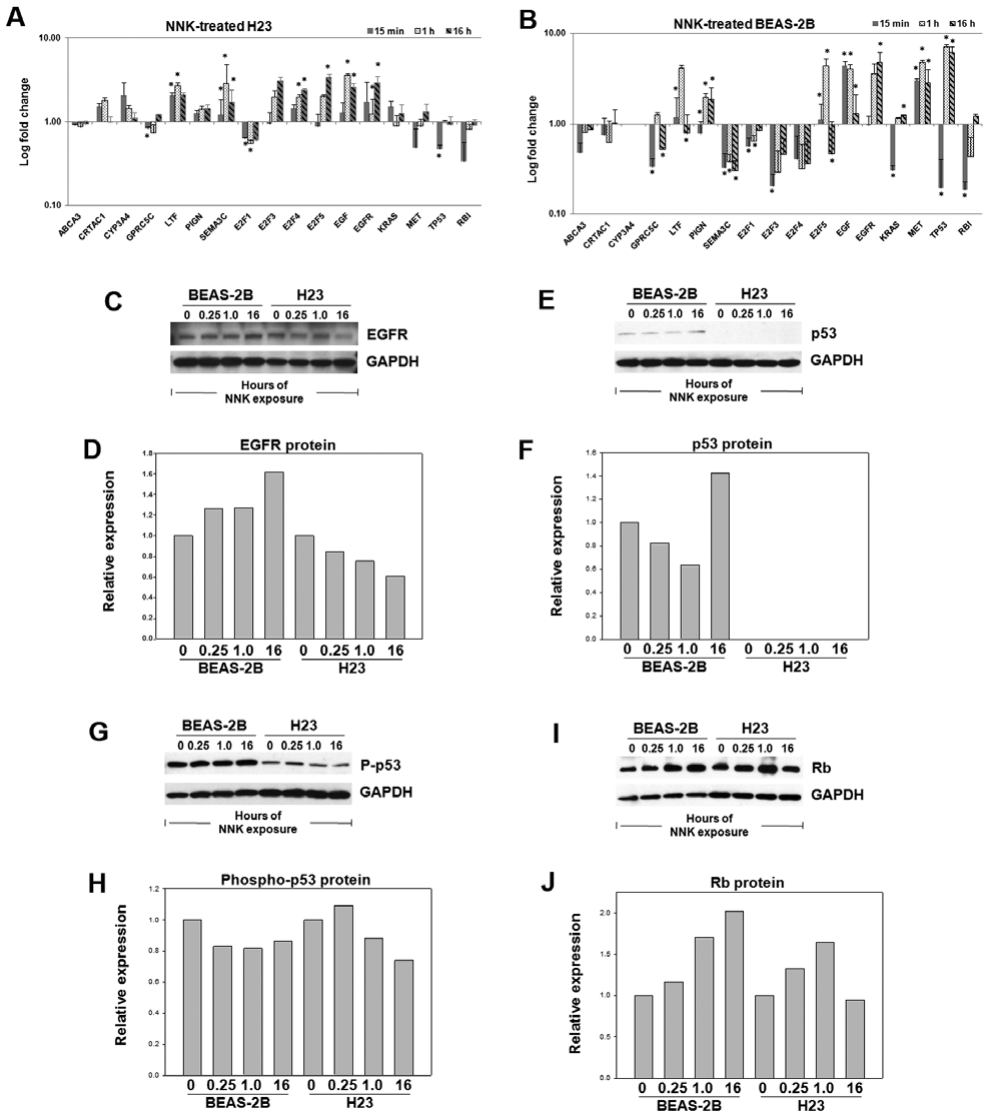
NNK-induced gene and protein expression in H23 and BEAS-2B. Gene expression fold change in cell lines treated with NNK (100 nM) vs. control in (A) human lung adenocarcinoma cells H23 and (B) normal lung epithelial cells BEAS-2B. The gene expression was normalized with endogenous control gene UBC. An asterisk above a bar indicates significant (P<0.05) differential expression in repeated ANOVA tests of five biological samples and three technical repeats in qRTPCR assays. Protein expression measured by western blots in NNK treated cell lines (C and D) BEAS-2B and H23 for EGFR, (E and F) p53, (G and H) phospho-p53 and (I and J) Rb. The protein expression was quantified with densitometry and normalized with endogenous control protein GAPDH in three biological repeats.

BEAS-2B and H23 cells were treated with NNK for validation of smoking-associated gene expression. Each cell line was exposed to NNK for 15 min, 1 and 16 h. Ten signaling pathway genes and 7 signature genes were examined. Results showed that 9 genes (*GPRC5C*, *LTF*, *SEMA3C*, *E2F1*, *E2F4*, *E2F5*, *EGF*, *EGFR* and *TP53*) exhibited significant differential expression in the NNK-treated H23 cells (Fig. 3A). In BEASE-2B cells, all genes except *CYP3A4* were expressed following NNK exposure, with 13 genes (*GPRC5C*, *LTF*, *PIGN*, *SEMA3C*, *E2F1*, *E2F3*, *E2F5*, *EGF*, *EGFR*, *KRAS*, *MET*, *TP53* and *RB1*) exhibiting significant differential expression (Fig. 3B).

To further evaluate the NNK-induced protein expression, western blot assays were performed in NNK-treated BEAS-2B and H23 cells after 15 min, 1 and 16 h exposures. The results show that *EGFR* had consistent overexpression at both the mRNA and protein levels over the time course in BEAS-2B cells after NNK treatment. In H23 cells, *EGFR* exhibited over-expression at the mRNA level; however, protein expression was downregulated following NNK exposure (Fig. 3C and D). These results indicate that in normal lung epithelial cells, the *EGFR* gene is overexpressed upon NNK treatment, consistent with previous findings (28); whereas in lung adenocarcinoma cells, the NNK-induced transcriptional and translational regulation of *EGFR* are not concordant at the same time points.

*p53* had consistent NNK-induced expression patterns at mRNA and protein levels, with short-term downregulation followed by upregulation in BEAS-2B cells. As H23 is *p53* deficient, *p53* protein was not expressed in these cells (Fig. 3E and F). Interestingly, downregulation of phospho-*p53* was consistently observed in both NNK-treated BEAS-2B and H23 cells (Fig. 3G and H), concordant with its mRNA and total protein expression. These results are consistent with the report that NNK induces damage in the *p53* gene (29).

In NNK-treated BEAS-2B cells, *Rb* gene expression was first significantly downregulated at 15 min, returned to its normal expression at 1 h, and then showed modest overexpression at 16 h; whereas the *Rb* protein showed a steady overexpression following the NNK treatment at all-time points. In NNK-treated H23 cells, both mRNA and protein expression of *Rb* were downregulated at all-time points (Fig. 3I and J). These results are consistent with the reported increased phosphorylation of the *Rb* Ser^795^ 6–15 h after NNK treatment of normal human bronchial epithelial cells (NHBE) and small airway epithelial cells (SAEC). This, in turn, promoted cells entering into the S phase (at 15–21 h) (30).

### Confirmation of smoking-associated gene coexpression network in lung adenocarcinoma

**Figure 4:**
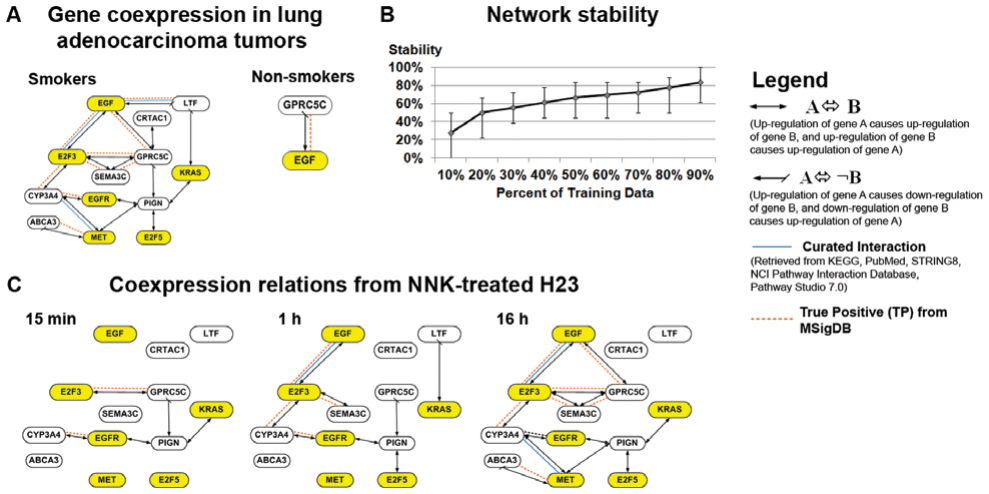
Smoking-associated coexpression network in lung adenocarcinoma. (A) Gene coexpression relations specific to smokers and non-smokers significant (P<0.05) in both training and test sets from Shedden *et al*(15) (network precision = 0.91, FDR = 0.01). (B) The stability of smoking-mediated networks as evaluated with random subsets of patients from the training cohort in 100 iterations. (C) Coexpression relations observed in the NNK-treated H23 cell line for 15 min, 1 and 16 h.

There were 17 gene coexpressions specifically associated with smokers, and one coexpression specifically associated with non-smokers significant (P<0.05) in both training and test sets from Shedden *et al*(15) (Fig. 4A). Among these 18 coexpression relations, 11 gene associations were confirmed with multiple biological databases (Fig. 4A; network precision=0.91; FDR<0.01), and the network structure was stable (Fig. 4B).

The smoking-associated gene coexpression network in lung adenocarcinoma patients (Fig. 4A) was further validated using the NNK-induced gene expression changes in lung adeno-carcinoma cell line H23 (Fig. 3A). Results show that most of the coexpressions in the smoking-associated network of lung adenocarcinoma tumors were confirmed by the coexpressions observed in NNK-treated H23 cells, at varying time points (Fig. 4C). All of the 17 smoking-associated gene coexpressions in NSCLC patients were observed in the cell experiments, except two coexpressions, one between *EGF* and *LTF* and one between *CRTAC1* and *GPRC5C*. These two unobserved gene coexpressions could be related to other sources of carcinogens in tobacco, because NNK is only one of the carcinogens in tobacco, among about 54 others (14). Overall, the smoking-associated gene coexpression network in NSCLC patients was largely confirmed in NNK-treated cell experiments, elucidating a network of smoking-induced gene alterations in NSCLC.

### Comparison with Bayesian belief networks and gene association networks based on Pearson’s correlation

This study presents novel implication network formalism for biomarker discovery. The ability to model cyclic relations in Genet overcomes the fundamental drawback of acyclic Bayesian networks in modelling molecular networks (31). In comparison with Bayesian belief networks, expression profiles of the identified 7 signature genes and 6 signalling pathway genes were used to build causal networks with TETRAD IV (http://www.phil.cmu.edu/projects/tetrad/current.html) for smoking and non-smoking lung adenocarcinoma patients in training and test sets, respectively (Fig. 2). There was only one interaction associated with smokers (between *MET* and *SEMA3C*; Fig. 2E) present in both training and test sets, which was considered a true positive when evaluated with MSigDB. In contrast, Genet generated significantly more biologically relevant gene coexpression relations that were validated by the biological databases and experimental results, confirming its topological advantage over the Bayesian belief networks.

Large-scale gene coexpression networks have been used in disease classification (32). These studies construct pair-wise gene coexpression networks by using correlation coefficients computed from gene expression profiles. Such networks indicate the distance or similarity between each pair of gene expression profiles but do not provide the direction or causal relations in the gene regulatory patterns. We have compared Genet with gene association networks based on Pearson’s correlation. In constructing smoking-mediated coexpression networks using 217 smoking and survival associated genes and 6 signalling hallmarks, both models had the same network precision and FDR. However, Genet generated significantly more biologically relevant gene association relations that were validated by the test set (20). These results indicate that prediction logic is more robust than Pearson’s correlation for inducting gene association networks.

## Discussion

In the United States, about 90% of male and 75–80% of female lung cancer deaths can be attributed to smoking each year (14). In recent years, lung adenocarcinoma, a rare tumor type in the early 20th century, has replaced squamous cell lung cancer as the most frequent cell type of NSCLC (33). The observations in the United States and abroad suggest that increases in lung adenocarcinoma cases since 1950 are more consistent with changes in smoking behavior and cigarette design than with diagnostic advances or histologic interpretation (34–36). The gene-smoking interactions and their relationship to lung cancer are not well established in epidemiology studies (14).

This study identified a 7-gene signature for lung cancer diagnosis and prognosis in smokers. The identified biomarker genes are involved in multiple lung cancer signaling pathways through concurrent coexpression with major signaling proteins. The 7-gene signature provided an accurate estimate of risk for tumor development and recurrence (as indicated by lung cancer survival) in smokers. The 7-gene signature also appeared to be a more accurate prognostic factor than commonly used clinicopathological factors for NSCLC. These results indicate the potential utility of this gene signature in predicting lung cancer risk in smokers before symptoms can be detected with morphological assessments in clinic. Such early detection could significantly improve the clinical outcome in lung cancer treatment. Furthermore, the 7-gene assay could potentially be used to identify specific patients at high-risk for tumor recurrence/metastasis using customized Affymetrix arrays, thus improving patient selection for adjuvant chemotherapy.

The gene expression-defined prognostic groups had a strong association with smoking and smoking cessation. Smokers were more likely to have the poor prognosis gene expression pattern than non-smokers. Furthermore, current smokers showed a stronger association with the poor prognosis gene expression pattern than previous smokers. These results suggest that the identified 7-gene signature is associated with smoking induced lung cancer initiation and progression, and the poor prognosis gene expression pattern might be reversed after smoking cession. Tobacco smoke contains a substantial amount of NNK, and the lowest dose shown to induce lung cancer in animal studies is remarkably close to the total dose of exposure experienced by a smoker in their lifetime (37). The smoking-associated gene coexpression network computationally derived from NSCLC patient transcriptional profiles was confirmed in the NNK-treated H23 cell line, further attesting to its biological relevance and smoking association in lung cancer.

Using the same methodology, a 6-gene (20) and an 8-gene signature were also identified from 217 smoking and survival associated genes, by modeling concurrent coexpression with different sets of 6 signaling hallmarks randomly selected from 10 KEGG human NSCLC signaling pathways (Table III). These 10 signaling proteins were selected based on their reported clinical relevance in NSCLC. The prognostic performance of the 6- and 8-gene signatures was comparable with the 7-gene signature (20) (Fig. 1). The 6- and 7-gene signatures both outperformed the clinicopathological covariates, but the 8-gene signature did not (results not shown). There is one common gene, *SEMA3C*, between the 6- and 7-gene signatures. In the experimental validation, all 10 signaling pathway genes showed significant differential expression in NNK treated normal lung epithelial cells and lung adenocarcinoma cells. The observed NNK-induced protein expression of *p53*, phospho-*p53*, *Rb* and *EGFR* was largely concordant with their mRNA expression levels in the BEAS-2B normal lung epithelial cells. In lung adenocarcinoma cell line H23, the NNK-induced gene expression was concordant with protein expression of *p53*, phospho-*p53* and *Rb*, but not of *EGFR*. These results indicate that *p53*, *Rb* and *EGFR* might be functionally involved in smoking-induced lung cancer initiation and progression. *EGFR* mutations, associated with better chemoresponse, are significantly associated with non-smokers compared to smokers in a large epidemiology study (38). The identified gene signatures were concurrently coexpressed with these signaling pathways in patient transcriptional profiles. The association of these gene signatures with smoking, smoking cessation, as well as lung cancer risk and survival, in turn, supports the involvements of these oncoproteins in smoking induced lung cancer initiation and progression.

## Figures and Tables

**Table I:** Summary of clinical characteristics of patients from the Director’s Challenge Study (15).

	UM and HLM (training set)		MSK and DFCI (testing set)	
	Smokers	Non-smokers	Smokers	Non-smokers
Patient sample size	149	20	151	29
Age (mean, s.d.)	65 (10)	68 (11)	63 (10)	66 (11)
Gender (male %)	54	0	48	31
Median survival (mo)	42	54	48	43
Tumor stage (%)				
I	58	80	65	55
II	22	5	25	28
III	18	10	10	17
Unknown	1	5	0	0

**Table II:** The identified smoking-associated 7-gene signature.

Gene symbol	Gene title	Molecular function (Gene Ontology)
ABCA3	ATP-binding cassette, sub-family A (ABC1), member 3	ATP, nucleotide binding; ATPase, transporter activity
CRTAC1	Cartilage acidic protein 1	Calcium ion binding
CYP3A4	Cytochrome P450, family 3, subfamily A, polypeptide 4	Monooxygenase, electron carrier, oxidoreductase activity; heme, metal ion and steroid binding
GPRC5C	G protein-coupled receptor, family C, group 5, member C	Receptor activity; protein binding
LTF	Lactotransferrin	Ferric iron, heparin, metal ion, protein binding; peptidase, serine-type endopeptidase activity
PIGN	Phosphatidylinositol glycan anchor biosynthesis, class N	Phosphotransferase, transferase activity
SEMA3C	Sema domain, immunoglobulin domain (Ig), short basic domain, secreted, (semaphorin) 3C	Receptor activity; semaphorin receptor binding

**Table III:** Multivariate Cox proportional analysis of the 7-gene risk score and major clinical covariates in smoking lung cancer patients from the test cohort (MSK and DFCI) in Director’s Challenge Study (15).

Variable^a^	P-value	Hazard ratio (95% CI)^b^
Analysis without 7-gene risk score		
Gender (male)	0.55	1.17 (0.70, 1.95)
Age at diagnosis (>60)	0.35	1.31 (0.74, 2.29)
Tumor differentiation		
Moderately differentiated	0.30	0.63 (0.26, 1.51)
Poorly differentiated	0.89	1.06 (0.47, 2.38)
Cancer stage		
II	1.54E-03	2.60 (1.44, 4.71)
III	5.53E-05	4.48 (2.16, 9.29)
Analysis with 7-gene risk score		
Gender (male)	0.51	1.19 (0.71, 1.99)
Age at diagnosis (>60)	0.49	1.22 (0.69, 2.16)
Tumor differentiation		
Moderately differentiated	0.33	0.65 (0.27, 1.55)
Poorly differentiated	0.93	0.96 (0.43, 2.16)
Cancer stage		
II	1.64E-03	2.61 (1.44, 4.74)
III	3.29E-05	4.79 (2.29, 10.04)
7-gene risk score	0.03	1.89 (1.06, 3.38)

a Gender was a binary variable (0 for female and 1 for male); age at diagnosis was a binary variable (0 for <60-year-old and 1 otherwise); tumor grade was categorical variable of 3 categories [well (as the reference group), moderately and poorly differentiated]; tumor stage was categorical variable of 3 categories [stage I (as the reference group), stage II and stage III].

b Denotes confidence interval.

## References

[1] Christiani DC (2006). Genetic susceptibility to lung cancer. J Clin Oncol.

[2] Spira A, Beane JE, Shah V (2007). Airway epithelial gene expression in the diagnostic evaluation of smokers with suspect lung cancer. Nat Med.

[3] Massion PP, Zou Y, Chen H (2008). Smoking-related genomic signatures in non-small cell lung cancer. Am J Respir Crit Care Med.

[4] Woenckhaus M, Klein-Hitpass L, Grepmeier U (2006). Smoking and cancer-related gene expression in bronchial epithelium and non-small-cell lung cancers. J Pathol.

[5] Guo NL, Tosun K, Horn K (2009). Impact and interactions between smoking and traditional prognostic factors in lung cancer progression. Lung Cancer.

[6] Beer DG, Kardia SL, Huang CC (2002). Gene-expression profiles predict survival of patients with lung adenocarcinoma. Nat Med.

[7] (2009). General Thoracic Surgery.

[8] Hung RJ, McKay JD, Gaborieau V (2008). A susceptibility locus for lung cancer maps to nicotinic acetylcholine receptor subunit genes on 15q25. Nature.

[9] Mani KM, Lefebvre C, Wang K (2008). A systems biology approach to prediction of oncogenes and molecular perturbation targets in B-cell lymphomas. Mol Syst Biol.

[10] Cui Q, Ma Y, Jaramillo M (2007). A map of human cancer signaling. Mol Syst Biol.

[11] Guo NL, Wan YW, Bose S (2011). A novel network model identified a 13-gene lung cancer prognostic signature. Int J Comput Biol Drug Des.

[12] Wan YW, Beer DG, Guo NL (2012). Signaling pathway-based identification of extensive prognostic gene signatures for lung adenocarcinoma. Lung Cancer.

[13] Schuller HM (2002). Mechanisms of smoking-related lung and pancreatic adenocarcinoma development. Nat Rev Cancer.

[14] Hecht SS (1999). Tobacco smoke carcinogens and lung cancer. J Natl Cancer Inst.

[15] Shedden K, Taylor JM, Enkemann SA (2008). Gene expression-based survival prediction in lung adenocarcinoma: a multi-site, blinded validation study. Nat Med.

[16] Raponi M, Zhang Y, Yu J (2006). Gene expression signatures for predicting prognosis of squamous cell and adenocarcinomas of the lung. Cancer Res.

[17] Bild AH, Yao G, Chang JT (2006). Oncogenic pathway signatures in human cancers as a guide to targeted therapies. Nature.

[18] Li C (2008). Automating dChip: toward reproducible sharing of micro-array data analysis. BMC Bioinformatics.

[19] Hildebrand DK, Laing JD, Rosenthal H (1977). Prediction Analysis of Cross Classifications.

[20] Guo NL, Wan YW (2012). Pathway-based identification of a smoking associated 6-gene signature predictive of lung cancer risk and survival. Artif Intell Med.

[21] Maglott D, Ostell J, Pruitt KD, Tatusova T (2007). Entrez Gene: gene-centered information at NCBI. Nucleic Acids Res.

[22] Ogata H, Goto S, Sato K (1999). KEGG: Kyoto Encyclopedia of Genes and Genomes. Nucleic Acids Res.

[23] Jensen LJ, Kuhn M, Stark M (2009). STRING 8 - a global view on proteins and their functional interactions in 630 organisms. Nucleic Acids Res.

[24] Subramanian A, Tamayo P, Mootha VK (2005). Gene set enrichment analysis: A knowledge-based approach for interpreting genome-wide expression profiles. Proc Natl Acad Sci USA.

[25] Ucar D, Neuhaus I, Ross-MacDonald P (2007). Construction of a reference gene association network from multiple profiling data: application to data analysis. Bioinformatics.

[26] Livak KJ, Schmittgen TD (2001). Analysis of relative gene expression data using real-time quantitative PCR and the 2(-Delta Delta C(T)) method. Methods.

[27] Witten IH, Frank E (2005). Data Mining: Practical Machine Learning Tools and Techniques.

[28] Lonardo F, Dragnev KH, Freemantle SJ (2002). Evidence for the epidermal growth factor receptor as a target for lung cancer prevention. Clin Cancer Res.

[29] Cloutier JF, Drouin R, Weinfeld M, O’Connor TR, Castonguay A (2001). Characterization and mapping of DNA damage induced by reactive metabolites of 4-(methylnitrosamino)-1-(3-pyridyl)-1-butanone (NNK) at nucleotide resolution in human genomic DNA. J Mol Biol.

[30] Ho YS, Chen CH, Wang YJ (2005). Tobacco-specific carcinogen 4-(methylnitrosamino)-1-(3-pyridyl)-1-butanone (NNK) induces cell proliferation in normal human bronchial epithelial cells through NFkappaB activation and cyclin D1 up-regulation. Toxicol Appl Pharmacol.

[31] Sachs K, Perez O, Pe’er D, Lauffenburger DA, Nolan GP (2005). Causal protein-signaling networks derived from multiparameter single-cell data. Science.

[32] Choi JK, Yu U, Yoo OJ, Kim S (2005). Differential coexpression analysis using microarray data and its application to human cancer. Bioinformatics.

[33] Travis WD, Travis LB, Devesa SS (1995). Lung cancer. Cancer.

[34] Ernster VL (1994). The epidemiology of lung cancer in women. Ann Epidemiol.

[35] Levi F, Franceschi S, La Vecchia C, Randimbison L, Te VC (1997). Lung carcinoma trends by histologic type in Vaud and Neuchatel, Switzerland, 1974–1994. Cancer.

[36] Thun MJ, Lally CA, Flannery JT (1997). Cigarette smoking and changes in the histopathology of lung cancer. J Natl Cancer Inst.

[37] Hoffmann D, Rivenson A, Hecht SS (1996). The biological significance of tobacco-specific N-nitrosamines: smoking and adenocarcinoma of the lung. Crit Rev Toxicol.

[38] Ren JH, He WS, Yan GL (2012). EGFR mutations in non-small-cell lung cancer among smokers and non-smokers: a meta-analysis. Environ Mol Mutagen.

